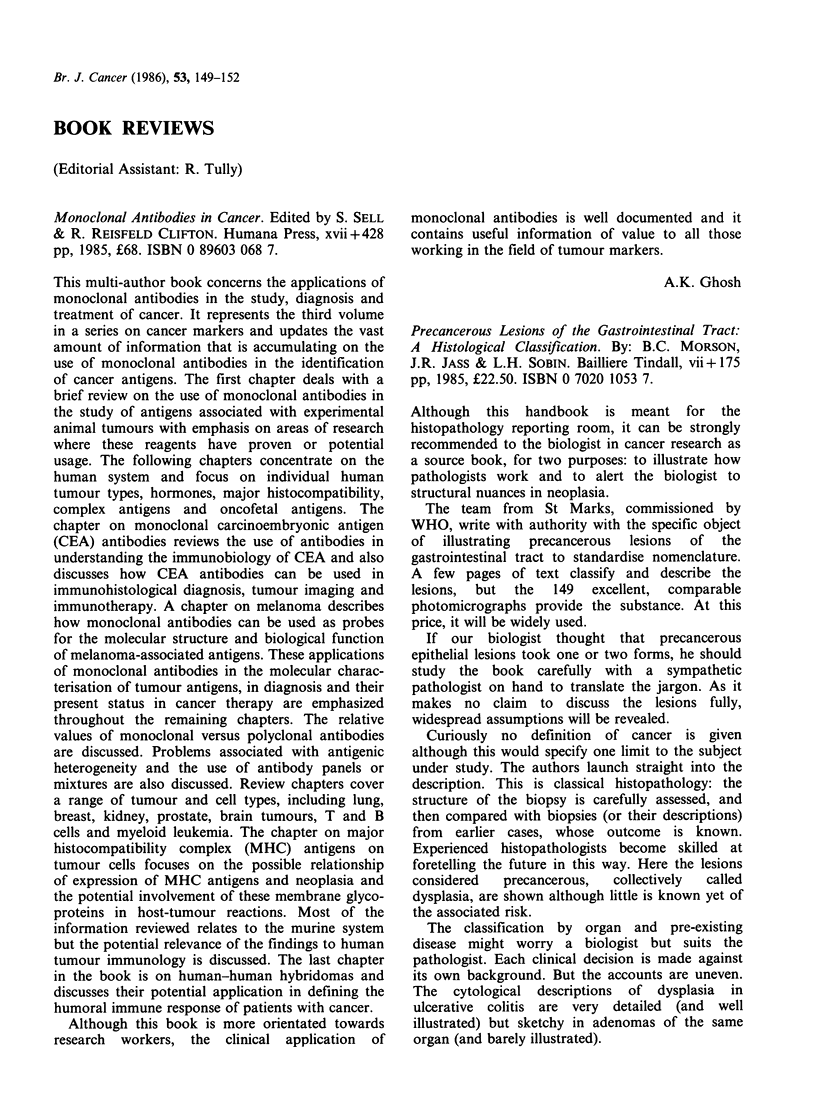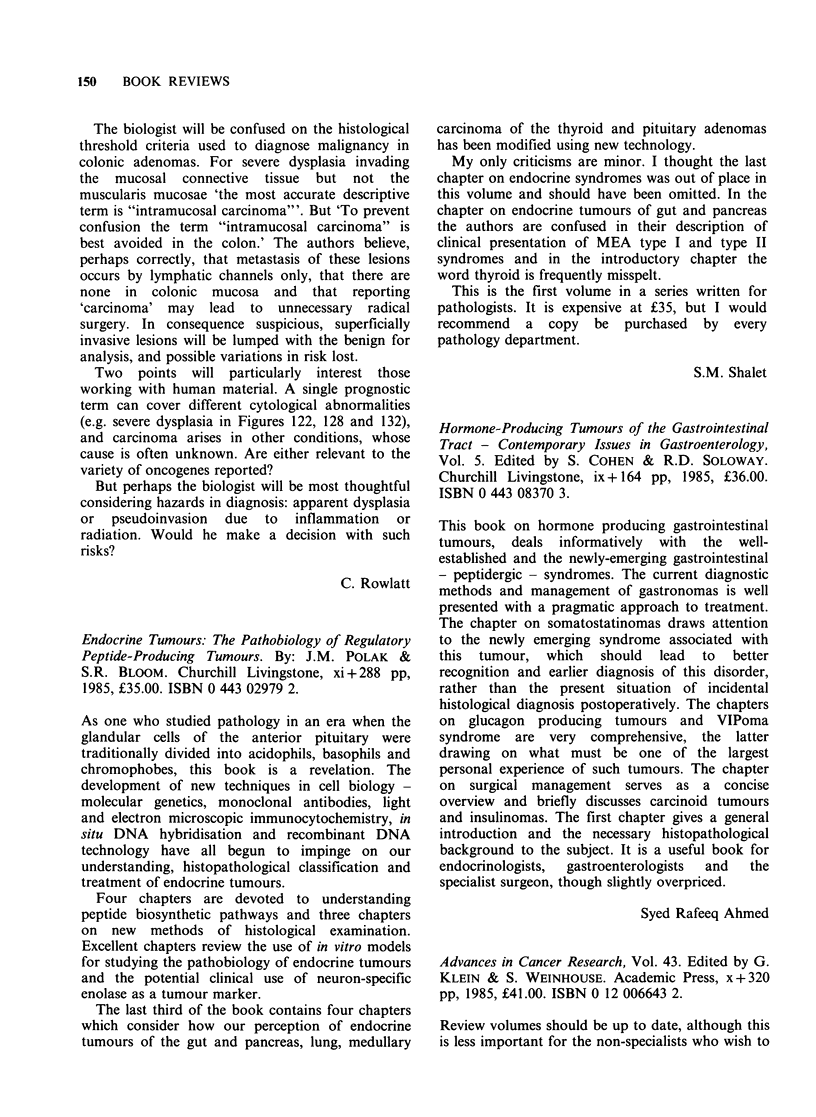# Precancerous Lesions of the Gastrointestinal Tract: A Histological Classification

**Published:** 1986-01

**Authors:** C. Rowlatt


*Precancerous Lesions of the Gastrointestinal Tract: A Histological Classification*. By: B.C. MORSON, J.R. JASS & L.H. SOBIN. Bailliere Tindall, vii+175 pp, 1985, £22.50. ISBN 0 7020 1053 7.

Although this handbook is meant for the histopathology reporting room, it can be strongly recommended to the biologist in cancer research as a source book, for two purposes: to illustrate how pathologists work and to alert the biologist to structural nuances in neoplasia.

The team from St Marks, commissioned by WHO, write with authority with the specific object of illustrating precancerous lesions of the gastrointestinal tract to standardise nomenclature. A few pages of text classify and describe the lesions, but the 149 excellent, comparable photomicrographs provide the substance. At this price, it will be widely used.

If our biologist thought that precancerous epithelial lesions took one or two forms, he should study the book carefully with a sympathetic pathologist on hand to translate the jargon. As it makes no claim to discuss the lesions fully, widespread assumptions will be revealed.

Curiously no definition of cancer is given although this would specify one limit to the subject under study. The authors launch straight into the description. This is classical histopathology: the structure of the biopsy is carefully assessed, and then compared with biopsies (or their descriptions) from earlier cases, whose outcome is known. Experienced histopathologists become skilled at foretelling the future in this way. Here the lesions considered precancerous, collectively called dysplasia, are shown although little is known yet of the associated risk.

The classification by organ and pre-existing disease might worry a biologist but suits the pathologist. Each clinical decision is made against its own background. But the accounts are uneven. The cytological descriptions of dysplasia in ulcerative colitis are very detailed (and well illustrated) but sketchy in adenomas of the same organ (and barely illustrated).

The biologist will be confused on the histological threshold criteria used to diagnose malignancy in colonic adenomas. For severe dysplasia invading the mucosal connective tissue but not the muscularis mucosae 'the most accurate descriptive term is "intramucosal carcinoma"'. But 'To prevent confusion the term "intramucosal carcinoma" is best avoided in the colon.' The authors believe, perhaps correctly, that metastasis of these lesions occurs by lymphatic channels only, that there are none in colonic mucosa and that reporting 'carcinoma' may lead to unnecessary radical surgery. In consequence suspicious, superficially invasive lesions will be lumped with the benign for analysis, and possible variations in risk lost.

Two points will particularly interest those working with human material. A single prognostic term can cover different cytological abnormalities (e.g. severe dysplasia in Figures 122, 128 and 132), and carcinoma arises in other conditions, whose cause is often unknown. Are either relevant to the variety of oncogenes reported?

But perhaps the biologist will be most thoughtful considering hazards in diagnosis: apparent dysplasia or pseudoinvasion due to inflammation or radiation. Would he make a decision with such risks?

C. Rowlatt